# Methylation Motifs in Promoter Sequences May Contribute to the Maintenance of a Conserved ^m5^C Methyltransferase in *Helicobacter pylori*

**DOI:** 10.3390/microorganisms9122474

**Published:** 2021-11-30

**Authors:** Bowen Meng, Naomi Epp, Winsen Wijaya, Jan Mrázek, Timothy R. Hoover

**Affiliations:** 1 Department of Microbiology, University of Georgia, Athens, GA 30602, USA; bmeng@uga.edu (B.M.); neepp@bethelks.edu (N.E.); Winsen.Wijaya@uga.edu (W.W.); 2 Department of Microbiology and Institute of Bioinformatics, University of Georgia, Athens, GA 30602, USA; mrazek@uga.edu

**Keywords:** DNA methylation, *Helicobacter pylori*, 5-methylcytosine DNA methyltransferase

## Abstract

DNA methylomes of *Helicobacter pylori* strains are complex due to the large number of DNA methyltransferases (MTases) they possess. *H. pylori* J99 M.Hpy99III is a 5-methylcytosine (^m5^C) MTase that converts GCGC motifs to G^m5^CGC. Homologs of M.Hpy99III are found in essentially all *H. pylori* strains. Most of these homologs are orphan MTases that lack a cognate restriction endonuclease, and their retention in *H. pylori* strains suggest they have roles in gene regulation. To address this hypothesis, green fluorescent protein (GFP) reporter genes were constructed with six putative promoters that had a GCGC motif in the extended −10 region, and the expression of the reporter genes was compared in wild-type *H. pylori* G27 and a mutant lacking the M.Hpy99III homolog (M.HpyGIII). The expression of three of the GFP reporter genes was decreased significantly in the mutant lacking M.HpyGIII. In addition, the growth rate of the *H. pylori* G27 mutant lacking M.HpyGIII was reduced markedly compared to that of the wild type. These findings suggest that the methylation of the GCGC motif in many *H. pylori* GCGC-containing promoters is required for the robust expression of genes controlled by these promoters, which may account for the universal retention of M.Hpy99III homologs in *H. pylori* strains.

## 1. Introduction

*Helicobacter pylori*, a member of the phylum Campylobacterota, which was formerly known as the subphylum Epsilonproteobacteria [1], colonizes the stomach of about half the human population worldwide [2,3]. Infection of the gastric mucosa by *H. pylori* is the major factor for peptic ulcer disease and chronic gastritis, and a major risk factor for gastric cancer and mucosa-associated lymphoid tissue lymphoma [4,5,6]. Several factors facilitate host colonization by *H. pylori*, including urease, catalase, and motility [7,8,9].

A remarkable feature of *H. pylori* genomes is the large number of genes encoding the DNA restriction–modification (R–M) systems as compared to the genomes of other bacteria [10,11,12]. There are four types of R–M systems, with Type II R–M systems being the simplest and most prevalent in bacteria [12]. Type II R–M systems consist of a restriction endonuclease and MTase that act independently of each other. The restriction endonuclease recognizes and cuts a defined DNA motif, while the MTase methylates a specific nucleotide within the motif to prevent the restriction of the bacterium’s genomic DNA. The primary and major role of bacterial R–M systems is to protect the genome of the cell by the restriction of incoming foreign DNA [13], although bacterial MTases have additional roles in gene expression, DNA replication, cell cycle control, and chromosome maintenance [14,15]. *H. pylori* is naturally competent, which may account for the high number of R–M systems in *H. pylori* as this could serve to protect the bacterium from invading foreign DNA [16]. Although most MTases are associated with a restriction enzyme, some MTases in *H. pylori* and other bacteria are orphan MTases that lack cognate restriction enzymes.

MTases catalyze the addition of a methyl group from the donor S-adenosyl methionine to adenine or cytosine. Three types of DNA methylation have been identified in bacteria: *N*^6^-methyladenine (^m6^A), *N*^4^-methylcytosine (^m4^C), and ^m5^C [17]. Methylomes of *H. pylori* strains are highly diverse due to the different sets of MTases within a given strain [10,18,19]. An examination of base modifications to the *H. pylori* UM032 genome revealed 17 methylated sequence motifs [19], while methylome analyses of *H. pylori* strains 26695 and J99-R3 identified 17 and 22 methylated sequence motifs, respectively [10]. Some *H. pylori* MTase genes are phase-variable due to the presence of homopolymeric nucleotide repeats, which are susceptible to length changes resulting from slipped-strand mispairing [10,20]. Despite the variability of *H. pylori* methylomes, a few target motifs appear to be methylated in all or nearly all *H. pylori* strains based on a study by Vale and co-workers where they examined genomic DNA from 221 *H. pylori* strains for susceptibility to cleavage by 29 methylation-sensitive restriction enzymes [21].

The MTase M.Hpy99III from *H. pylori* J99 methylates GCGC sequences in order to generate G^m5^CGC motifs, and based on a gene sequence analysis, M.Hpy99III homologs were predicted to be present and active in all 459 *H. pylori* strains examined by Estibariz and co-workers [22]. The gene encoding the cognate restriction endonuclease for M.Hpy99III (*jhp1049*) was found in only 61 of the 459 *H. pylori* strains analyzed, but only 15 of these genes were predicted to be functional, and the rest were pseudogenes with premature stop codons and/or frameshift mutations [22]. The presence of *jhp1049* homologs among *H. pylori* strains displays a biased phylogeographic distribution, as a majority of the strains that carry the gene belong to a population with substantial African ancestry [22].

A transcriptome analysis of *H. pylori* J99, *H. pylori* BCM-300, and mutants of the two strains where the gene encoding the M.Hpy99III homolog was inactivated showed that the loss of the MTase resulted in the altered expression of 225 genes in J99 and 29 genes in BCM-300, 10 of which were down-regulated in both mutant strains [22]. The growth rate as well as the expression of genes involved in important phenotypic traits, including adherence to host cells, natural competence, cell shape, and copper sensitivity, were affected in the *H. pylori* J99 M.Hpy99III mutant [22]. Taken together, these observations suggest that the methylation of GCGC sequences by M.Hpy99III plays an important role in the regulation of gene expression in *H. pylori*.

In contrast to mammalian systems, where the role of ^m5^C in gene expression is well characterized, little is known about how ^m5^C influences gene expression in bacteria. *H. pylori* M.HpyAVIBM is an orphan ^m5^C MTase that methylates CCTC sequences to generate ^m5^CCTC motifs [23,24]. Deleting genes that encode the homologs of M.HpyAVIBM in the *H. pylori* strains AM5 and SS1 affected the expression of a number of genes with roles in motility, adhesion, and virulence, although for some genes, the loss of the ^m5^C MTase had opposing effects on expression in the two strains [25]. *Escherichia coli* K-12 strains possess an orphan ^m5^C MTase Dcm that recognizes the sequence CCWGG. The loss of Dcm results in the increased expression of several categories of genes [26,27,28]. Dcm has been proposed to repress the expression of *rpoS*, and the loss of Dcm results in increased levels of RpoS, which leads to the elevated expression of RpoS-dependent genes [26]. The loss of the orphan ^m5^C MTase VchM in *Vibrio cholerae* results in an attenuated growth, both in vivo in infant mice and in vitro in competition with wild-type cells [29]. Transcriptome analysis identified 134 genes with elevated transcript levels in a Δ*vchM* mutant compared to the wild-type strain, but only about half of these genes contained the methylation target sequence [29]. Moreover, mutating sites methylated by VchM in three of the four genes up-regulated in the Δ*vchM* mutant had no significant effect on transcript levels, although mutating all three motifs in one gene did result in a ~5-fold increase in transcript abundance [29]. These observations indicate that while ^m5^C may directly influence the expression of some genes in *V. cholerae*, many of the genes whose expression was altered in the Δ*vchM* mutant are indirectly affected by ^m5^C.

In the case of *H. pylori* M.Hpy99III, Estibariz and co-workers identified a GCGC motif in the extended −10 region of the *jhp0832* promoter that apparently needs to be methylated for optimal promoter activity [22]. In the present study, we analyzed the frequency of GCGC motifs in the *H. pylori* 26695 genome and the positional distribution of GCGC motifs within predicted promoter regions. A strong bias for the GCGC motif near the −13 position relative to the transcriptional start site (TSS) was observed. Expression of GFP reporter genes under the control of various *H. pylori* GCGC-containing promoters identified three promoters where the methylation of the GCGC motif appeared to be required for robust promoter activity. Taken together, these findings suggest that the methylation of the GCGC motif has a functional role in many GCGC-containing promoters, and that the GCGC motifs have been maintained by selective pressure.

## 2. Materials and Methods

### 2.1. Bacterial Strains and Growth Conditions

*E. coli* DH5α was used for cloning and plasmid construction. *E. coli* strains were grown in LB broth or agar medium supplemented with kanamycin (30 μg/mL) or ampicillin (100 μg/mL) when appropriate. For routine growth of *H. pylori* strains, the cultures were grown microaerobically under an atmosphere consisting of 10% CO_2_, 4% O_2_, and 86% N_2_ at 37 °C on tryptic soy agar (TSA) supplemented with 5% heat-inactivated horse serum. When required, erythromycin (100 μg/mL) or kanamycin (30 μg/mL) was added into the *H. pylori* growth medium.

For growth rate determinations, *H. pylori* strains grown for 3–4 days on TSA, as described above, were used to inoculate 15 mL of Brain Heart Infusion (BHI) supplemented with 5% heat-inactivated horse serum in a 100 mL serum vial with a glass tube sidearm that fit into a Klett colorimeter. The cells were grown overnight at 37 °C in the serum vials under an atmosphere consisting of 5% CO_2_, 10% H_2_, 10% O_2_, and 75% N_2_, and then sub-cultured the next day into 15 mL of fresh medium and grown under the same conditions. Klett readings for each culture were recorded at various times and used to generate growth curves. At least three biological replicates for each strain were used to calculate the growth rates, and a two-tailed Student’s *t*-test was used to determine statistical significance.

### 2.2. Inactivation of Genes Encoding M.HpyGIII H. pylori G27 and M.HpyB128II in H. pylori B128

M.HpyGIII (encoded by *hpg27_1066*) and M.HpyB128II (encoded by *hpb128_202g26*) are orthologs of M.Hpy99III in *H. pylori* G27 and *H. pylori* B128, respectively. The genes encoding these MTases were disrupted with an erythromycin-resistant cassette (*ermB*) as follows: iProof DNA polymerase (Bio-Rad, Hercules, CA, USA) was used for PCR with genomic DNA (gDNA) from *H. pylori* B128 purified using the Wizard^®^ Genomic DNA Purification Kit (Promega, Madison, WI, USA) as the template. Sequences of primers used for PCR are indicated in Table S1. The primer set hp1121up_F2 and hp1121up_R2 was used to amplify a 505-bp DNA fragment that included 413 bp of DNA upstream of the predicted start codon of *hpB128_202g26*, plus 92 bp of the *hpb128_202g26* coding sequence. The primer set hp1121d_F2 and hp1121d_R2 was used to amplify a 576-bp DNA fragment that included 489 bp of DNA downstream of the stop codon *hpb128_202g26*, plus 87 bp of the coding sequence. The primer set ermB_OLF and ermB_OLR was used to amplify an erythromycin-resistant cassette from pSB167 [30]. The primers ermB_OLF and hp1121up_R2 shared a complementary sequence, as did the primers ermB_OLR and hp1121d_F2, and the three amplicons were joined by overlapping the PCR. The resulting amplicon was transformed into *H. pylori* B128, and erythromycin-resistant transformants were selected on TSA supplemented with 5% heat-inactivated horse serum and erythromycin. The insertion of *ermB* into *hpb128_202g26* was confirmed by PCR using the hp1121up_F2 and hp1121d_R2 primer set and the subsequent DNA sequencing of the resulting amplicon. The same amplicon was transferred into *H. pylori* G27 via natural transformation to disrupt *hpg27_1066*. PCR and DNA sequencing confirmed the replacement of *hpg27_1066* with the *ermB* cassette in the *H. pylori* G27 chromosome.

### 2.3. Construction of gfp Reporter Genes

Synthetic genes containing the coding region of *gfp,* in which the codon usage was optimized for *H. pylori,* was synthesized by Genewiz (South Plainfield, NJ, USA) (Figure S3). In addition to the *gfp* coding region, the synthetic genes contained the *H. pylori ureA* ribosome-binding site and the promoter regions of *H. pylori* G27 *hpg27_846*, *hpg27_24* (*icd*), or *hpg277_1129* (*cah*), which were flanked by XhoI and NheI restriction sites. Sequences of the promoter regions spanned positions −65 to +10 relative to the TSSs that were predicted using a TSS database generated from the *H. pylori* 26695 transcriptome [31]. A BamHI restriction site was introduced immediately downstream of the *gfp* stop codon in the synthetic gene. The synthetic genes, which were provided by the supplier in plasmid pUC57-Kan, were cloned into the XhoI and BamHI restriction sites in the shuttle vector pHel3 [32] to generate plasmids pECBM27, pECBM28, and pECBM29. To construct additional *gfp* reporter genes, the DNA corresponding to the promoter regions were amplified by PCR using iProof DNA polymerase (Bio-Rad) and gDNA from either *H. pylori* J99 (for *gfp* reporter gene constructs with the predicted primary promoter of *jhp0160* or putative antisense promoter internal to *jhp0334* (*kgtP*)) or *H. pylori* G27 (for putative antisense promoter internal to *hpg27_865*) as the template. Primers used for PCR are listed in Table S1. Each primer contained an XhoI or NheI restriction site to facilitate the exchange of the *hpg27_846* promoter in the plasmid pECBM27 with the new promoter region. For the construction of the *jhp0160*-*gfp* and *kgtP*-*gfp* reporter genes, ~320 bp DNA fragments were amplified from *H. pylori* J99 gDNA, which corresponded to position +4 relative to the predicted TSS, up to ~300 bp upstream of the GCGC motif. For the construction of the *hpg27_865*-*gfp* reporter gene, a DNA fragment corresponding to positions +6 to −56 relative to the predicted TSS was amplified from the *H. pylori* G27 gDNA. Promoter regions of each reporter plasmid were sequenced (Eton Biosciences, San Diego, CA, USA) to confirm that the plasmid was correct. 

### 2.4. gfp Reporter Gene Assays

*H. pylori* cells were grown on TSA medium supplemented with kanamycin, and after 4–5 days, the cells were harvested and resuspended in phosphate buffer (pH 8.0, 15 mM KH_2_PO_4_ + 17 mM K_2_HPO_4_) to a final OD_600_ of 0.5. Samples were placed into in a Bio-One CELLSTAR^TM^ 96-well polystyrene round-bottom cell culture microplate (Greiner, Frickenhausen, Germany). A Biotek Synergy^TM^ Mx Microplate Reader (Winooski, VT, USA) was used to detect fluorescence at 462 nm excitation and 510 nm absorbance, with a gain of 75. Three biological replicates and technical replicates were used to collect data for each reporter gene. Statistical analysis of the data was performed using an unpaired *t*-test with GraphPad Prism 9.0.2 (GraphPad Software, Inc., San Diego, CA, USA). 

### 2.5. Restriction Digestion Analyses

Genomic DNA from *H. pylori* strains were harvested from plates using the Wizard Genomic DNA Purification Kit (Promega) after being grown for 4–5 days. Following DNA extraction, 1 μg of gDNA was digested with HinP1I (New England Biolabs, Ipswich, MA, USA) for 1 h and the resulting digestion reaction was run out on a 0.8% agarose gel.

### 2.6. Bioinformatic Analysis

Computer simulations were used to assess the occurrence of the promoter motif under the conditions of a null hypothesis, which assumed that the motif was not subject to direct selective constraints but was potentially influenced by other biases, including a biased codon and amino acid usage, dinucleotide usage biases, or local variance in GC content. For the simulation, 1000 randomized genomes were generated from the *H. pylori* 26695 genome using the “m1c1” model in Genome Randomizer [33]. In the modeling, the genome was separated into segments consisting of protein-coding genes and intergenic regions. Each intergenic region was modeled as a first-order Markov chain using the nucleotide alphabet, and each gene as a first-order Markov chain using the codon alphabet, where the next codon probability depended on the last base of the previous codon. Consequently, the model reproduced the compositional heterogeneity of the sequence at the scale of individual genes (e.g., GC-rich genes or intergenic segments in the AT-rich *H. pylori* genome retain their lower AT content) as well as the asymmetry between the sense (coding) and antisense (template) strands, and between the leading and lagging strands with respect to the direction of replication (i.e., GC-skew). Moreover, the model reproduced the dinucleotide frequencies in each intergenic region and codon frequencies as well as the frequencies of dinucleotides spanning adjacent codons in each gene.

Motif Locator (https://www.cmbl.uga.edu//software/motloc.html; date accessed 23 June 2020) was used to identify potential GCGC-containing promoters in the *H. pylori* J99 genome. The sequences of 76 potential GCGC-containing promoters identified from a transcriptome analysis of *H. pylori* 26695 [31] were aligned with the GCGC motif as the reference point. The sequences were 51 nucleotides in length and corresponded to the TSS identified in the transcriptome analysis along with 50 nucleotides of the sequence upstream of the TSS [31]. Motif Locator converted the alignment into a position-specific score matrix (PSSM), then scanned the *H. pylori* J99 genome sequence for all words of 51 nucleotides in length with a PSSM score higher than a given cutoff, *S*_0_. The default score cutoff, which was the tenth percentile among all scores of the 78 motif sequences in the alignment, was used for the analysis. The primary output of the program was a set of coordinates in the analyzed DNA sequence of motifs, similar to those in the alignment. These coordinates could be subsequently passed to other programs (*r*-scan statistics, pattern vicinity analysis) to provide additional information about the distribution of the matching motifs in the analyzed sequence and with respect to genes.

## 3. Results

### 3.1. Comparison of H. pylori 26695 Promoter Sequences Containing GCGC Motifs

**Figure 1:**
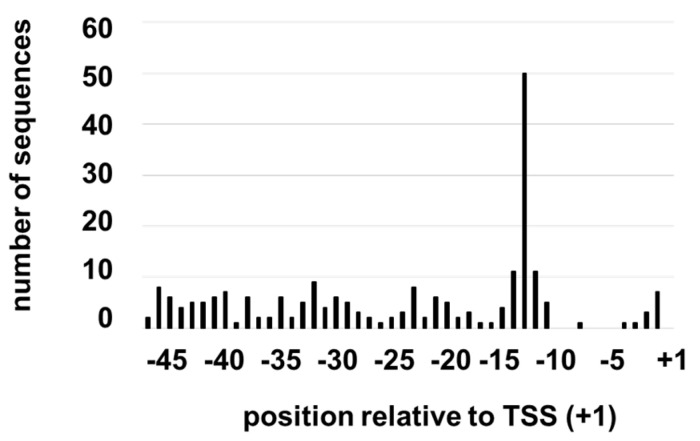
Positional distribution of GCGC motifs in *H. pylori* 26695 promoter regions. The position of the 3′-end of the GCGC motif relative to the TSS was determined for 211 putative promoter sequences that contain a GCGC motif within 50 nucleotides of the TSS. There were 217 non-overlapping GCGC motifs and 7 overlapping GCGC motifs (i.e., GCGCGC) in the promoter sequences.

A search in a TSS database for the *H. pylori* 26695 transcriptome [31] revealed 211 putative promoters with a GCGC motif within 50 nucleotides of the TSS (Table S1). About 39% of the sequences were predicted primary or secondary promoters of genes, ~14% were within coding regions of genes and in the same orientation (internal promoters), while ~47% were within coding regions but in the opposition orientation (antisense promoters). Positions of the GCGC motifs within the promoter regions were not distributed uniformly as there was a marked preference around position −13 (measured from the 3′-end of the GCGC motif) (Figure 1). GCGC motifs appeared to be distributed randomly across the rest of the promoter region, with the exception of the area spanning positions −5 to −10 for which there was a single GCGC motif in the 211 promoter sequences. The excess of GCGC counts at position −13 (50 GCGC motifs) relative to any other position in the promoter region was highly significant, with *p*-value < 0.00001 using a two-tail binomial test. The bias against GCGC motifs in the −5 to −10 region may be a consequence of a strong preference for A and T residues in the region from −7 to −12 of all putative promoters in the *H. pylori* 26695 TSS database [31] (Figure S1). Our further investigation focused on the subset of *H. pylori* promoters with a GCGC motif in the −13 region, which we hereafter refer to as GCGC-containing promoters.

**Figure 2:**
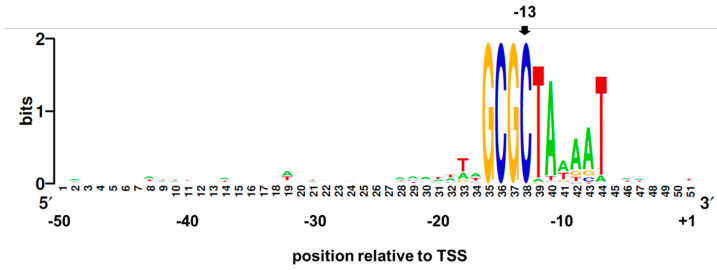
Conservation of *H. pylori* 26695 promoters with a GCGC motif near position −13. Promoter sequences were inferred from the TSS database reported by Sharma and co-workers [31]. The 3′-ends of the GCGC motifs in the promoters were positioned from −11 to −15 relative to the TSS, with −13 being the most common position. The GCGC motifs were aligned for 79 promoter sequences and from the alignment a sequence logo was generated using WebLogo (https://weblogo.berkeley.edu/logo.cgi; date accessed 25 June 2020) [39].

Sequences of the GCGC-containing promoters were compared to determine if they shared sequence similarity. Since the distance between core promoter elements and the TSS may vary due to various factors, including promoter sequence, DNA topology, and concentrations of initiation nucleotide triphosphates [34,35,36,37,38], we aligned the sequences of all the promoters where the 3′-end of the GCGC motif was positioned from −11 to −15 relative to the predicted TSS (Table S2). A sequence alignment of the 79 GCGC-containing promoters revealed that the nucleotides at positions −7 (T), −11 (A), and −12 (T) were highly conserved, and also indicated a weaker preference for A at positions −8 and −9 (Figure 2). These preferences for A and T in the −7 to −12 region are a general characteristic of *H. pylori* promoters, including those not containing the GCGC motif (Figure S1).

### 3.2. Activities of Some GCGC-Containing Promoters Are Inhibited in H. pylori G27 Mutant Lacking M.HpyGIII

A previous study of the *H. pylori* J99 *jhp0832* promoter suggested that the methylation of a GCGC motif in the extended −10 region of the promoter was required for robust promoter activity [22]. To expand upon this previous study, we constructed green fluorescent protein (*gfp*) reporter genes with six potential GCGC-containing promoters from the *H. pylori* strains G27 and J99, and examined the expression of the *gfp* reporter genes in wild-type *H. pylori* G27 and a mutant strain in which *hpg27_1066* (encodes the M.Hpy99III ortholog M.HpyGIII) was inactivated with a cassette containing an erythromycin-resistant marker. We chose to analyze putative promoters from *H. pylori* J99 and G27 since these strains were commonly used for molecular genetic studies, and we wished to evaluate the efficacy of using putative promoter sequences from *H. pylori* 26695 in identifying potential promoters in these strains. Each *gfp* reporter gene carried the ribosome-binding site (RBS) from *ureA*, which was placed immediately upstream of the *gfp* coding sequence since two of the promoters examined were potential antisense promoters and lacked an associated RBS. The *H. pylori* G27 promoters that were examined included the predicted primary promoters for *icdA* (isocitrate dehydrogenase), *cah* (α-carbonic anhydrase), and *hpg27_846*, which corresponded to the *H. pylori* J99 *jhp0832* promoter examined by Estibariz and co-workers [22]. A fourth *H. pylori* G27 promoter examined was a potential antisense promoter in *hpg27_865* that corresponded to an antisense GCGC-containing promoter in *hp0914* [31]. The two remaining *gfp* reporter genes were constructed with predicted GCGC-containing promoters of genes that were differentially expressed in the *H. pylori* J99 M.Hpy99III mutant as compared to the wild type [22]. One of the J99 promoters was the putative primary promoter for *jhp0160*, which was down-regulated in the M.Hpy99III mutant [22]. The other J99 promoter was a potential antisense promoter in the coding sequence of *kgtP* (encodes α-ketoglutarate permease), which was up-regulated in the M.Hpy99III mutant [22].

The *gfp* reporter genes were introduced into wild-type *H. pylori* G27 and a *hpg27_1066* mutant on the shuttle vector pHel3. The restriction enzyme HinP1I recognizes and cleaves the motif 5′-GCGC-3′, but is sensitive to CpG methylation. To confirm if M.HpyGIII was functional, gDNA from the wild type and the *hpg27_1066* mutant were digested with the restriction enzyme HinP1I, which cut the unmethylated GCGC motifs in the *hpg27_1066* mutant gDNA but not the methylated GCGC sites in the wild-type gDNA (Figure S2). Disrupting *hpg27_1066* interfered with the growth of *H. pylori* G27, as the doubling time of the *hpg27_1066* mutant (8.6 ± 1.8 h) was significantly slower than that of the wild-type parental strain (4.1 ± 0.94 h; *p*-value < 0.05). Disrupting the M.Hpy99III homolog in *H. pylori* B128 (M.HpyB128II) similarly allowed for digestion of gDNA with HinP1I (Figure S2), but loss of the MTase did not significant impair the growth of *H. pylori* B128 (Appendix A).

**Figure 3:**
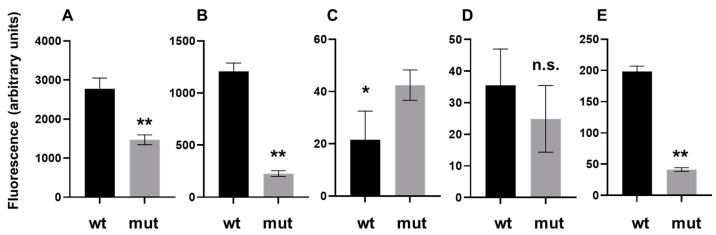
Expression of GFP reporter gene bearing putative GCGC-containing promoters in *H. pylori* G27 wild type and mutant lacking M.HpyGIII. (**A**) GFP reporter gene containing the *H. pylori* G27 *icdA* promoter; (**B**) GFP reporter gene containing the *H. pylori* G27 *hpg27_846* promoter; (**C**) GFP reporter gene containing the *H. pylori* G27 *cah* promoter; (**D**) GFP reporter gene containing a predicted antisense promoter within *H. pylori* G27 *hpg27_865*; (**E**) GFP reporter gene containing *H. pylori* J99 *jhp0160*. Mean values for GFP fluorescence in wild-type *H. pylori* G27 are indicated by black bars, while mean values for GFP fluorescence in the *H. pylori* G27 mutant lacking M.HpyGIII are shown in gray bars. Six to twelve replicates were prepared for each condition. Error bars indicate 95% confidence intervals. Statistical analysis of the data was performed using an unpaired *t*-test with GraphPad Prism 9.0.2. Asterisks indicate differences in GFP fluorescence that were significant (**, *p*-value < 0.0001; *, *p*-value = 0.0014).

The P*_icdA_-gfp* and P*_hpg27_846_-gfp* reporter genes were expressed at high levels in the wild-type strain, while the expression levels of the reporter genes were reduced ~2-fold and ~4-fold, respectively, in the *hpg27_1066* mutant (Figure 3A,B). In contrast to the P*_icdA_-gfp* and P*_hpg27_846_-gfp* reporter genes, the P*_cah_-gfp* reporter gene was expressed at a low level, and the expression of the reporter gene was slightly higher in the *hpg27_1066* mutant (Figure 3C). The expression of the reporter gene bearing the predicted antisense promoter within the *hpg27_865* coding sequence (asP*_hpg27__865*) was low, and there was no significant difference in expression between the wild-type and *hpg27_1066* mutant strains (Figure 3D). The putative antisense promoter from the *kgtP* coding sequence (asP*_kgtP_*) did not appear to be functional since neither wild-type nor *hpg27_1066* mutant cells harboring the asP*_kgtP_-gfp* reporter gene exhibited fluorescence above background levels. The P*_jhp0160_-gfp* reporter gene was expressed moderately well, but at a lower level compared to the P*_icdA_-gfp* and P*_hpg27_846_-gfp* reporter genes, and the expression of the P*_jhp0160_-gfp* reporter gene was reduced ~7-fold in the *hpg27_1066* mutant as compared to the wild type (Figure 3E).

The wide variation in the activities of the promoters tested in the *gfp* reporter gene assays was likely due to differences in the nucleotide sequences of the promoters. To address this issue, we compared the sequences of the predicted promoters for the 29 genes reported to be down-regulated in the *H. pylori* BCM-300 M.Hpy99III mutant [22] in order to identify the DNA elements that may be important for a robust methylation-dependent promoter activity. Using DNA sequences from the *H. pylori* 26695 TSS database [31], we identified ten potential primary GCGC-containing promoters associated with down-regulated genes in the *H. pylori* BCM-300 MTase mutant, which could account for transcriptional control of 19 of the 29 down-regulated genes in the mutant (Table S1). We hypothesized that the GCGC motifs in these ten GCGC-containing promoters needed to be methylated for optimal promoter activities. Aligning the sequences of these promoters revealed highly conserved features (Figure S3). The sequence logo for the GCGC-containing promoters from *H. pylori* BCM-300 was similar to that of the larger set of GCGC-containing promoters from *H. pylori* 26695 (Figure 2), with the notable difference being the greater AT-richness of the 5-nucleotide sequence immediately upstream of the GCGC motif in the *H. pylori* BCM-300 promoter sequences. In addition, nucleotides that were well conserved in the sequence alignment of the larger set of GCGC-containing promoters (positions −7, −11, and −12) were absolutely conserved in the *H. pylori* BCM-300 promoter set.

Table 1 shows the sequences of the GCGC-containing promoters that were used to construct the *gfp* reporter genes. All the promoters tested in the *gfp* reporter gene assays had the T residue at position −7, which was absolutely conserved in the *H. pylori* BCM-300 promoter set (Figure S3). With the exception of the *jhp0160* promoter, all the promoters tested in the *gfp* reporter gene assays also had the conserved TA dinucleotide at positions −12 and −11, whereas the *jhp0160* promoter had a TT dinucleotide. Thus, the large differences in expression levels of the various *gfp* reporter genes cannot be attributed directly to elements in the −7 to −12 region of the promoters. The promoter elements present in *gfp* reporter genes that were expressed robustly (P*_icdA_-gfp*, P*_hp0846_-gfp,* and P*_jhp0160_-gfp*), but absent in the promoters of *gfp* reporter genes that were expressed poorly, included a TT dinucleotide immediately upstream of the GCGC motif and an A located nine nucleotides upstream of the GCGC motif (Table 1). Both of these DNA elements were conserved in the *H. pylori* BCM-300 promoter set (Figure S3).

### 3.3. Potential GCGC-Containing Promoters within Protein-Coding Regions May Have Arisen by Chance in the Absence of Selection

A striking observation from our initial search in the *H. pylori* 26695 TSS database was that ~60% of the putative promoters that had a GCGC motif near the TSS were internal or antisense promoters (Table S1). Several recent studies have raised questions about the roles of intragenic promoters [40,41,42,43,44,45]. While many intragenic promoters are verifiably active [36,37,38,39,40,41], their physiological roles are often obscure. A combination of experiments and computational simulations suggested that promoters as well as transcription factor binding sites in general are likely to arise by chance even in the absence of selection, which could be beneficial in promoting evolutionary adaptations by increasing the dynamics of evolution of gene regulatory networks. To investigate whether the internal and antisense GCGC-containing promoters in *H. pylori* may have arisen by chance in the absence of selection, we used the model described by Mrázek and Karls [46].

**Figure 4:**
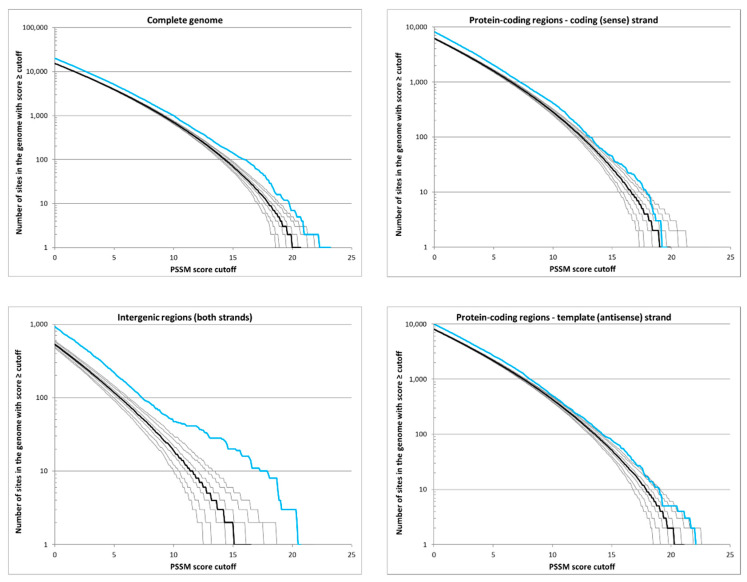
Reverse cumulative distributions of PSSM scores for motifs that resemble GCGC-containing promoters in the *H. pylori* 26695 genome. The blue line represents the observed values for the *H. pylori* 26695 genome, and the black lines represent the random sequences. The y-axis indicates the number of sites in the genome with scores greater than or equal to the PSSM score cutoff indicated on the x-axis. The thick black line represents the median for 1000 random sequences, and the thin black lines correspond to the 90th, 95th, and 99th percentiles.

Comparisons of PSSM score distributions using the PSSM derived from the alignment of the GCGC-containing promoters in the *H. pylori* 26695 genome and 1000 randomized genomes are shown in Figure 4. Only the right tail of the distribution with PSSM scores > 0 is shown. For PSSM scores close to zero, the simulations were expected to match the observed values because such low scores typically do not indicate active promoters. This was true for most transcription factors investigated by Mrázek and Karls [46]. However, the values for the predicted GCGC-containing promoters were systematically lower for the random sequences than for the actual *H. pylori* genome (Figure 4). This indicates that even the more complex “m1c1” model used to generate the random sequences, which reproduces the codon usage, dinucleotide content, and sequence heterogeneity of the actual genome at the scale of individual genes, is not an accurate representation of the null hypothesis that would ideally consider all biases affecting the genome sequence, except those resulting directly from the selection on active GCGC-containing promoters. Nevertheless, the model matches the overall shape of the distribution of PSSM scores in the genome up to the PSSM value of ~11, after which the deviation from the random sequences begins to increase. This is clearly visible in the intergenic sequences where physiologically important promoters are most likely to occur, and the increasing deviation from the random model at the higher PSSM score values is indicative of a number of active GCGC-containing promoters maintained by selection. In the absence of selection, the curve for the actual genome would be expected to match approximately the shape of the curve for the random sequences. This was the case for sequences from the protein-coding regions, as the values for the model and actual genome tended to converge for higher PSSM score values, with the exception of four potential promoters in the antisense strand with unexpectedly high PSSM scores > 20. The four potential antisense promoters with unexpectedly high PSSM scores were located in *bcp* (HP0136, encodes bacterioferritin co-migratory protein), *lpxL* (HP0280, encodes lipid A acyl transferase; [47]), *valS* (HP1153, encodes valyl-tRNA synthetase), and *alaS* (HP1241, encodes alanyl-tRNA synthetase). The TSS data for *H. pylori* 26695 [31] confirmed the sequences in *lpxL*, *valS,* and *alaS* as functional antisense promoters, although the promoter in the coding sequence of *valS* was reported to be the primary promoter for the sRNA encoded in HPnc6160.

To evaluate the efficacy of the PSSM scores in identifying GCGC-containing sequences with potential promoter activity, we used the program Motif Locator (https://www.cmbl.uga.edu//software.html; date accessed 15 July 2020) to generate a PSSM from the alignment of the GCGC-containing promoters in the *H. pylori* 26695 genome. The PSSM was used by the program to scan the *H. pylori* 26695 genome sequence for all words of 51 nucleotides in length, with a PSSM score higher than the default score cutoff, which was set at 13.148, the tenth percentile of all scores for the motifs in the training set. The Motif Locator analysis identified 287 sequences that met or exceeded this cutoff score in the *H. pylori* 26695 genome, which was well above the number of sequences in the training set and indicated that only ~24% of the identified sequences appeared to be active promoters under the conditions used for the *H. pylori* 26695 transcriptome analysis [30]. Approximately 62% of the motifs with high PSSM scores that were located in intergenic regions and in the correct orientation to function as a primary or secondary promoter appeared to be active promoters (Table 2). In contrast, only ~19% of the motifs with high PSSM scores that were located in protein-coding regions appeared to be active promoters (Table 2). These data suggest that most sequences with high PSSM scores that are located in protein-coding regions are not active promoters maintained by selection, whereas sequences with high PSSM scores that are positioned in intergenic regions have a much higher probability of being active promoters.

## 4. Discussion

As demonstrated by Estibariz and co-workers, orthologs of the ^m5^C MTase M. Hpy99III play a critical role in *H. pylori* biology, as loss of the MTase in the *H. pylori* strains J99 and BCM-300 resulted in the altered expression of several genes in the two strains, many of which are involved in cellular functions required for host colonization [22]. While the altered expression of some of these genes was likely due to indirect effects, results from the study by Estibariz and co-workers indicated that the GCGC motif in the extended −10 region of the *jhp0832* promoter apparently needs to be methylated for optimal promoter activity [22]. To ascertain the distribution of GCGC motifs within *H. pylori* promoter regions, we searched in a dataset of probable promoter sequences for *H. pylori* 26695 generated in a transcriptome analysis [31]. Approximately 11% of the promoters in *H. pylori* 26695 (211 of 1914) possessed a GCGC motif within 50 nucleotides of the TSS. The GCGC motif showed a strong bias for the −13 region (Figure 1), suggesting that the positioning of the GCGC motif in this location has functional significance and has been maintained in the *H. pylori* 26695 genome by selective pressure.

Building upon the previous study of the *jhp0832* GCGC-containing promoter [22], we examined the expression of *gfp* reporter genes bearing one of six putative GCGC-containing promoters in wild-type *H. pylori* G27 and a mutant strain that lacked M.HpyGIII (Figure 3). Four of the promoters examined were primary promoters, while the other two were antisense promoters. Robust expression was observed for *gfp* reporter genes bearing primary promoters from three genes (*icdA*, *hpg27_846,* and *jhp0160*), and the expression of these reporter genes was reduced from 2-fold to 7-fold in the mutant strain that lacked M.HpyGIII as compared to the wild type (Figure 3). We infer from these results that the optimal activity of these three promoters is dependent on the methylation of the GCGC motifs within the promoters. The fourth primary promoter examined in the *gfp* reporter gene assays was that from *cah*, and in contrast to the P*_icdA_*-*gfp*, P*_hpg27_*__846_-*gfp,* and P*_jhp0160_*-*gfp* reporter genes, the P*_cah_*-*gfp* reporter gene was expressed at a low level, and the expression of the reporter gene was ~2-fold higher in the MTase mutant (Figure 3C). The assay results for the P*_cah_-gfp* reporter gene differed from the report that *cah* transcript levels were reduced from 2-fold to 3-fold in *H. pylori* J99 and BCM-300 MTase mutants as compared to the wild-type parental strains [22]. The low activity of the *H. pylori* G27 *cah* promoter may have contributed to the discrepancy in the results of the P*_cah_*-*gfp* reporter gene assay and the previous transcriptome analysis. Similar to the P*_cah_*-*gfp* reporter gene, the expression of the *gfp* reporter genes bearing putative antisense promoters was either very low or failed to exceed background levels. It is possible that the P*_cah_*-*gfp*, asP*_hpg27_865_*-*gfp,* and asP*_kgtP_*-*gfp* reporter genes are missing *cis*-acting elements that are needed for transcription initiation, which may account for the low expression levels of these *gfp* reporter genes. Alternatively, these promoters may be intrinsically weak.

An interesting question is—how does the methylation of the GCGC motif influence promoter activity? The methylation of GCGC motifs may influence the activity of GCGC-containing promoters by affecting base readout in the DNA major groove or the shape readout in the DNA minor groove [48], thereby causing an impact on interactions between RNA polymerase and the promoter. Each nucleotide base pair has a unique array of group signatures in the major groove that can be recognized by specific amino acid residues in protein–DNA interactions. The methylation of C-5 in cytosine alters the functional group signature in the major groove by replacing a nonpolar hydrogen with a bulkier and more hydrophobic methyl group. The 5-methyl group in ^m5^C and thymine is situated at the major groove edge, and the ^m5^C-G base pair may be contacted through hydrophobic interactions, similar to how thymine is contacted. If the methyl group of ^m5^C and thymine is sufficient to confer binding specificity, then in principle, the two nucleotides should be able to substitute for each other in DNA-binding sites. In studies with synthetic *E. coli lac* operators, the substitution of a specific A-T base pair with a G-C base pair, but not a G-^m5^C base pair, decreased the stability of the LacI–operator complex [49]. Replacing the A-T base pair with an A-U base pair also reduced the stability of the LacI–operator complex, which implicates the thymine 5-methyl group as the primary functional group recognized by LacI at this base pair since the A-U base pair has the same group signature as the A-T base pair, with the exception of the 5-methyl group [49].

Since the *H. pylori* GCGC-containing promoters have well-conserved elements (Figure 2 and Figure S3), we reasoned that the conserved sequences could be leveraged to accurately predict GCGC-containing promoters in the genomes of other *H. pylori* strains or even other *Helicobacter* species. To evaluate the efficacy of the approach, we used PSSM generated from a training set of GCGC-containing promoter sequences to search the *H. pylori* 26695 genome and then compare the search results with identified promoter sequences in the *H. pylori* 26695 genome [31]. Using the PSSM for intergenic sites produced a reasonably acceptable false positive rate (~38%), but the false positive rate for sequences within protein-coding regions was very high (~81%) (Table 2). One possible explanation for this discrepancy could be the contextual effects, such as the local DNA or nucleoid structure in the promoter region, which cannot be captured in the PSSM model and could be different for intergenic and intragenic promoters [50]. Comparing the sequences of GCGC-containing promoters with sequences that were not associated with identified promoters but had high PSSM scores failed to yield insight into DNA elements that may be important for promoter function. The large number of sequences with high PSSM scores that were not associated with putative promoters, particularly motifs that were located within protein-coding regions, suggest that most of these sites arise by chance in the absence of natural selection. Consistent with this hypothesis, PSSM score distributions using the PSSM derived from the alignment of the GCGC-containing promoters did not differ significantly between the *H. pylori* 26695 genome and randomized genomes for intragenic sequences (Figure 4). In contrast, the PSSM score distribution for intergenic sequences in the *H. pylori* 26695 genome deviated strongly from that of the randomized genomes when the PSSM value exceeded 11 (Figure 4), which is indicative of the maintenance of active GCGC-containing promoters within intergenic regions by selection.

Similar to the report with *H. pylori* J99 [22], the loss of the M.Hpy99III homolog (M.HpyGIII) in *H. pylori* G27 interfered with growth as the doubling time of the mutant was approximately twice that of the wild-type parental strain. We postulate that the decreased growth rates of the *H. pylori* G27 and J99 MTase mutants was due to the altered gene expression resulting from the decreased transcription initiation from one or more GCGC-containing promoters. Although the loss of the M.Hpy99III homolog in *H. pylori* BCM-300 did not alter the growth rate, it did inhibit the expression of genes involved in several important processes, including iron transport, vitamin biosynthesis, peptidoglycan biosynthesis, TCA cycle, sugar transport, and tRNA modification; the loss also affected copper sensitivity and cell viability [22]. The global effects of M.Hpy99III homologs on gene expression likely account for the retention of this MTase in *H. pylori* strains in the absence of the cognate restriction endonuclease. Alternatively, Estibariz and co-workers suggested that the methylation of GCGC motifs may influence global gene regulation by affecting DNA topology [22], which might also account for the retention of the MTase in *H. pylori* strains.

## Figures and Tables

**Table 1:** Sequences of predicted GCGC-containing promoters examined in *gfp* reporter gene assays.

Gene	Promoter Type	Promoter Sequence ^1^
*gfp* reporter genes expressed at moderate to high levels		
*icd*	primary	ATTCAAAAAAAAGATTTTTAGGGGTTATATAGTATTTTTGCGCTAGTATAGTTACTCA
*hpg27_846*	primary	TCCACTCAAACCCCTTATAACGCTTAAACCAAATCGCTTGCGCTATAATGAGCTGATA
*jhp0160*	primary	TCATATTGATAAGCTTACTATATAAGATTAAGGGACTTTGCGCTTAAATACCCCTTAA
*gfp* reporter genes expressed at undetectable to low levels		
*cah*	primary	ACCAATCTATTTTTTGTAACTGCGGTCATTGTTTATTGAGCGCTAGAATTAATTGCTT
*hpg27_865*	antisense	GTGAGGTTGGTCATCATGTAACTTCTGTCTTGGTAATTAGCGCTAAAATCGCCATCTG
*kgtP*	antisense	TGAAAAACCCTAGCATAAAAACTAAAAAAGCTGAGATGAGCGCTAGAGTAGGGTCATT

^1^ GCGC motifs of promoter sequences are aligned and in red. Nucleotides absolutely conserved in the down-regulated GCGC-containing proteins in the *H. pylori* BCM-300 MTase mutant (Table S1) are indicated in bold, and highly conserved nucleotides are underlined.

**Table 2:** Distribution of potential GCGC-containing promoters in the *H. pylori* 26695 genome predicted by the Motif Locator program.

Location/Orientation of Motifs	Coding Regions		Intergenic/Partial Overlap	
	Sense	Antisense	Sense	Antisense
number motifs	106	125	42	14
number motifs associated with TSS	18	25	26	1

## Data Availability

Data are available upon request.

## Supplementary Materials

The following are available online at https://www.mdpi.com/article/10.3390/microorganisms9122474/s1, Figure S1: Sequence log for putative *H. pylori* 26695 promoters, Figure S2: Digestion of gDNA from wild-type *H. pylori* G27 and B128, and mutants that lack the M.Hpy99III homolog, Figure S3: Sequence logo for GCGC-containing promoters in *H. pylori* BCM-300 that may require the methylation of the GCGC motif for optimal activity, Figure S4: Template used for constructing *gfp* reporter genes, Table S1: Sequences of primers used in this study, Table S2: Down-regulated gene in the *H. pylori* BCM-300 mutant lacking the M.Hpy99III homolog that had a putative GCGC-containing promoter.

## Appendix A

In addition to confirming M.HpyGIII was functional in *H. pylori* G27, we similarly verified that the M.Hpy99III homolog in *H. pylori* B128 (M.HpyB128II) was functional by digesting gDNA from wild-type *H. pylori* B128 and an *hpb128_202g26* (encodes M.HpyB128II) mutant with HinP1I. We observed that the gDNA from the *hpb128_202g26* mutant, not the wild type, was cut with the restriction enzyme (Figure S2). The growth rate of the *hpb128*_*202g26* mutant (6.6 ± 2.5 h) was somewhat slower than that of the wild type (4.1 ± 0.6 h), but this difference was not statistically significant (*p*-value > 0.5).
