# Supplementary material for: Methylation Motifs in Promoter Sequences May Contribute to the Maintenance of a Conserved m5C Methyltransferase in Helicobacter pylori
Source: Microorganisms. 2021 Nov 30;9(12):2474. doi: 10.3390/microorganisms9122474 (PMC8706393; doi:10.3390/microorganisms9122474)
Supplement: Supplementary file 1 [file microorganisms-09-02474-s001.zip › microorganisms-1458085-supplementary.pdf]

Supplemental information

Table S1. Sequences of primers used in this study.

Hp1121_up_F2	5′-GCCGATACTTGCTGTAATGGA-3′	Constructing PCR product for inactivation of M.*Hpy*99III
Hp1121_up_R2	5′-TTGCGATACCGTCGAGATGGCTTCATG ATTGATTTCTGC-3′	Constructing PCR product for inactivation of M.*Hpy*99III
Hp 1121_d_F2	5′-TACTAAAGGGAATGTAGATAAGCAAGC CATTTGCTATCCCAA-3′	Constructing PCR product for inactivation of M.*Hpy*99III
Hp 1121_d_R2	5′-GGGGAATGATTTGTTGGATGAG-3′	Constructing PCR product for inactivation of M.*Hpy*99III
ermB_OLF	5′-ATCTCGACGGTATCGCAA-3′	Constructing PCR product for inactivation of M.*Hpy*99III
ermB_OLR	5′-TTATCTACATTCCCTTTAGTA-3′	Constructing PCR product for inactivation of M.*Hpy*99III
HPG27_865Fwd	5′-TCGATCCTCGAGAGCGTGAGGTTGGTCATCATGTAA -3′	Constructing GFP reporter plasmid for HPG27_865
HPG27_865Rev	5′-TACATTGCTAGCACCCAGATGGCGATTTTAGCGCTA-3′	Constructing GFP reporter plasmid for HPG27_865
JHP_0334_GFP_Fwd	5′-TCCAGCCTCGAGCATAAAAACCTTTTTTACCATTCT-3′	Constructing GFP reporter plasmid for JHP_0334
JHP_0334_GFP_Rev	5′-TACATTGCTAGCCAATGACCCTACTCTAGCGCTCAT-3′	Constructing GFP reporter plasmid for JHP_0334
JHP_0160_GFP_Fwd	5′-TCCAGCCTCGAGATTTAAAGTGGGTGAAAAATGTTC-3′	Constructing GFP reporter plasmid for JHP_0160
JHP_0160_GFP_Rev	5′-TACATTGCTAGCTTTAAGGGGTATTTAAGCGCAAAG-3′	Constructing GFP reporter plasmid for JHP_0160

Table S2. Down-regulated genes in *H. pylori* BCM-300 mutant lacking M.Hpy99III homolog that have a putative GCGC-containing promotor.

Gene name	Locus tag		[a]Promoter sequence alignment (-50 to +1)
	[b]BCM-300	26695	
icdA	BCM_00032<br>BCM_00033	HP0027	AAAAAAAGATTTTTAAGGGTTATATAGTATTTTT**GCGC**TAGTATAGTTACT<br>AAAAAAAGATTTTTAAGGGTTATATAGTATTTTT**GCGC**TAGTATAGTTACT
[c]*prtC*	BCM_00173<br>BCM_00172	HP0169	TTTAGAGGGCGTTTTGATCGCTAAAGCTTAAGT**GCGC**TAAAATGATAGCCG<br>TTTAGAGGGCGTTTTGATTGCTAAAGCTTAAGT**GCGC**TAAAATGACAGCCG
mraY<br>*murD*	BCM_00513<br>BCM_00514	HP0493	TACTTTAACTCATGCTGATTTTAAGCGAGCCAATTT**GCGC**TACAATTTTCT<br>CTACCCTATTCATGCTATTTTTAAGTAAATCAATTT**GCGC**TACAATTTTCT
pepA	BCM_00591<br>BCM_00590<br>BCM_00589	HP0570	CATGCGTTAGAGTGGCTTAAAAAACACCCTTAT**GCGC**TAATATTACTATTA<br>CATGCGTTAGGGTGGCTTAAAAAACACCCTTAT**GCGC**TAATATTACTATTA
[d]*feoB*	BCM_00707	HP0687	GTCTCGCTTTTATTATATAACAATTAGTCTTATA**GCGC**TATAGTGCTAACT<br>GTCTCGCTTTTATTATATAACAATTAGTCTTATA**GCGC**TATAGTGCTGACT
[d]*moeB*	[d]BCM_00859<br>BCM_00860	HP0813	AGAGACCGCTATTCTAGTCAAAATCGCCTTATTTTT**GCGC**TATGATATTCA<br>TAGAAGCGCTATTCTAGTCAAAATCGCCTTATTTTT**GCGC**TATGATTTTCA
gluP	[d]BCM_01112<br>BCM_01111	HP1175	AATCAAGCGTCTTAAGGCAATTTAAAATAAAATA**GCGC**TAGCATGAACCCA<br>AATCAAGCATCTTAAGGCAATTTAAAATAAAATA**GCGC**TAGCATGACCCTA
[d]*cah*	BCM_01124	HP1186	ATCTGTTTTTTGTAACTGCGGTCATTGTTGATTAA**GCGC**TAAAATTAAGGC<br>ATCTATTTTTTGTAACTGCGGTCATTGTGGATTAG**GCGC**TAGAATTAATTG
[d]*trmU*	BCM_01276	HP1335	CAATGGTTAAAAAGGGTAGTTATGAATGGTTTTT**GCGC**TAGACTACAAGCC<br>AATGGTTAAAAAAAGGTGGTTATGAATGGTTTTT**GCGC**TAGACTACGAGCC
[d,e]*crdR*	BCM_01307<br>BCM_01306<br>[d]BCM_01305	HP1365	TTTTTCATAACCGCTTTTGGTATAAAGGCTATAGAATTA**GCGC**TACAATACCCCCA<br>TTTTTTATAACCGCTTTTAGTATAAAGGC-----AATTA**GCGC**TACAATACCCCCA

[a]Top sequence is putative promoter in *H. pylori* BCM-300 and bottom sequence corresponds to promoter in *H. pylori* 26695 predicted from TSS database [1]. GCGC motifs are indicated in boldface.

[b]Locus tag numbers are indicated for genes that appear to be in an operon and were down-regulated in *H. pylori* BCM-300 MTase mutant [2].

[c]Predicted promoter upstream of HP0170 and BCM_00174.

[d]Corresponding gene is down-regulated in *H. pylori* 99 M.Hpy99III mutant [2] and has a predicted GCGC-containing promoter.

[c]Predicted promoters for *H. pylori* BCM-300 and *H. pylori* J99 *crdR* have a 5-bp insertion compared to *H. pylori* 26695 *crdR* promoter.

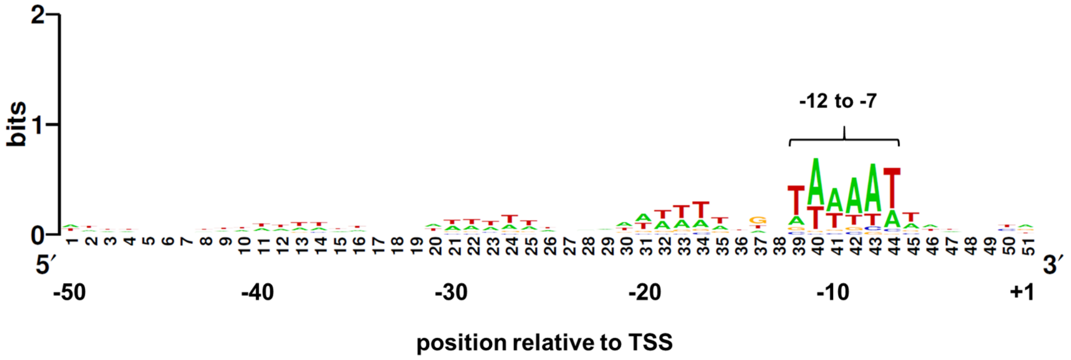

Figure S1. Sequence logo for putative *H. pylori* 26695 promoters. WebLogo was used to generate a sequence logo from an alignment of 1914 promoter sequences identified from a *H. pylori* 26695 transcriptome analysis [1]. The sequence logo shows a strong preference for A or T residues in positions -12 to -7.

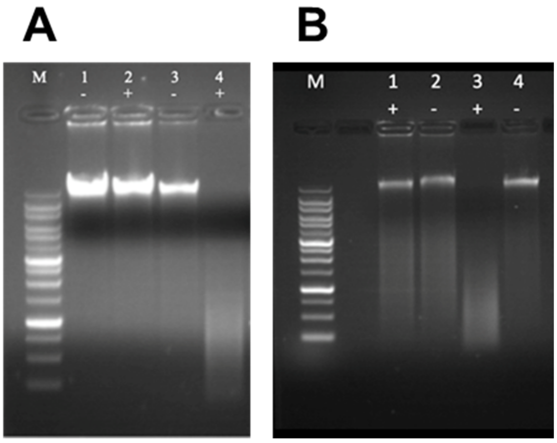

Figure S2. Digestion of gDNA from wild-type *H. pylori* G27 and B128 and mutants that lack the M.Hpy99III homolog. (**A**) DNA from wild-type *H. pylori* G27 (lanes 1 and 2) or the *hpg27_1066* mutant (lanes 3 and 4) was treated without (-) or with (+) HinP1I. (**B**) DNA from wild-type *H. pylori* B128 (lanes 1 and 2) or the *hpb128_202g26* mutant (lanes 3 and 4) was treated with (+) or without (-) HinP1I. Lane M contains 1kb PLUS™ DNA Ladder (GoldBio).

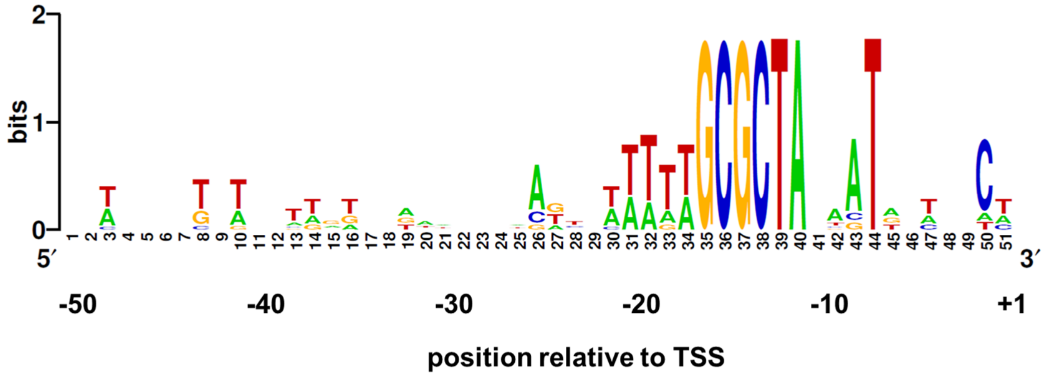

Figure S3. Sequence logo for GCGC-containing promoters in *H. pylori* BCM-300 that may require methylation of the GCGC motif for optimal activity. Promoter sequences were inferred from the TSS database reported by Sharma and co-workers [1]. The 3′-ends of the GCGC motifs in the promoters were positioned from -11 to -14 relative to the TSS. GCGC motifs of the 10 promoter sequences were aligned and a sequence logo was generated from the alignment with WebLogo [3].

A

XhoI *hpg27_846* promoter region
CTCGAGAGCTGTTTTAATCCACTCAAACCCCTTATAACGCTTAAACCAAATCGCTT**GCGC**TATAATGAGCTGATATTT
NheI *ureA* RBS start
TAAAGCTAGCAAGAATAGGAGAATGAG*ATG*GTGAGCAAAGGGGAAGAATTATTTACCGGGGTGGTGCCTATTTTAGTG
GAATTAGATGGGGATGTGAATGGGCATAAATTTAGCGTGAGCGGGGAAGGGGAAGGGGATGCTACCTATGGGAAATTA
ACCTTAAAATTTATTTGCACCACCGGGAAATTACCTGTGCCTTGGCCTACCTTAGTGACCACCTTAACCTATGGGGTG
CAATGCTTTAGCAGGTATCCTGATCATATGAAACAACATGATTTTTTTAAAAGCGCTATGCCTGAAGGGTATGTGCAA
GAAAGGACCATTTTTTTTTAAAGATGATGGGAATTATAAAACCAGGGCTGAAGTGAAATTTGAAGGGGATACCTTAGTG
AATAGGATTGAATTAAAAGGGATTGATTTTAAAGAAGATGGGAATATTTTAGGGCATAAATTAGAATATAATTATAAT
AGCCATAATGTGTATATTATGGCTGATAAACAAAAAAATGGGATTAAAGTGAATTTTAAAATTAGGCATAATATTGAA
GATGGGAGCGTGCAATTAGCTGATCATTATCAACAAAATACCCCTATTGGGGATGGGCCTGTGTTATTACCTGATAAT
CATTATTTAAGCACCCAAAGCGCTTTAAGCAAAGATCCTAATGAAAAAAGGGATCATATGGTGTTATTAGAATTTGTG
stop BamHI
ACCGCTGCTGGGATTACCTTAGGGATGGATGAATTATATAAA*TAA*GGATCC

B

XhoI *hpg27_24* (*icd*) promoter NheI
CTCGAGTTTAAACACGCATTCAAAAAAAAGATTTTTAGGGGTTATATAGTATTTTT**GCGC**TAGTATAGTTACTCAAATTTTAGCTAGC

C

XhoI *hpg27_1129* (*cah*) promoter NheI
CTCGAGTCCAACTTTTAACCAATCTATTTTTTGTAACTGCGGTCATTGTTTATTGA**GCGC**TAGAATTAATTGCTTATTAATAGCTAGC

Figure S4. Template used for constructing *gfp* reporter genes. (**A**) DNA sequence indicated above was synthesized by Genewiz and cloned into plasmid pUC57-Kan. Unique XhoI, NheI and BamHI restriction sites (underlined) were included to facilitate introduction of the reporter gene into other plasmids and swapping promoter regions. A 76-bp DNA sequence containing the *hpg27_846* promoter region and flanked by XhoI and NheI sites was included in the synthesized gene. Start and stop codons for *gfp* are indicated in italics. Codon usage of *gfp* was optimized for *H. pylori*. The ribosome binding site of *H. pylori ureA* (in red) was introduced between the NheI site and start codon of *gfp*. (**B**) A 76-bp DNA sequence containing the putative *H. pylori* G27 *icd* promoter that was synthesized and replaced the segment flanked by XhoI and NheI sites in the construct

shown in panel A. (**C**) A 77-bp DNA sequence containing the putative *H. pylori* G27 *cah* promoter that was synthesize and replaced the segment flanked by XhoI and NheI sites in the construct shown in panel A.

References

1. Sharma, C.M.; Hoffmann, S.; Darfeuille, F.; Reignier, J.; Findeiss, S.; Sittka, A.; Chabas, S.; Reiche, K.; Hackermuller, J.; Reinhardt, R.; et al. The primary transcriptome of the major human pathogen *Helicobacter pylori*. *Nature* **2010**, *464*, 250-255, doi:nature08756 [pii]
10.1038/nature08756.
2. Estibariz, I.; Overmann, A.; Ailloud, F.; Krebes, J.; Josenhans, C.; Suerbaum, S. The core genome m5C methyltransferase JHP1050 (M.Hpy99III) plays an important role in orchestrating gene expression in Helicobacter pylori. *Nucleic Acids Res* **2019**, *47*, 2336-2348, doi:10.1093/nar/gky1307.
3. Crooks, G.E.; Hon, G.; Chandonia, J.M.; Brenner, S.E. WebLogo: a sequence logo generator. *Genome research* **2004**, *14*, 1188-1190, doi:10.1101/gr.849004.
